# All-cause and cause-specific mortality of different migrant populations in Europe

**DOI:** 10.1007/s10654-015-0083-9

**Published:** 2015-09-11

**Authors:** Umar Z. Ikram, Johan P. Mackenbach, Seeromanie Harding, Grégoire Rey, Raj S. Bhopal, Enrique Regidor, Michael Rosato, Knud Juel, Karien Stronks, Anton E. Kunst

**Affiliations:** 1 Department of Public Health, Academic Medical Center, University of Amsterdam, P.O. Box 22660, 1100 DD Amsterdam, The Netherlands; 2 Erasmus University Medical Center, Rotterdam, The Netherlands; 3 King’s College, London, UK; 4 INSERM, CépiDc, Le Kremlin-Bicêtre, France; 5 University of Edinburgh, Edinburgh, UK; 6 Universidad Complutense de Madrid, Madrid, Spain; 7 Ulster University, Coleraine, Northern Ireland, UK; 8 University of Southern Denmark, Copenhagen, Denmark

**Keywords:** All-cause mortality, Cause-specific mortality, Migrant health, Migrants, Europe

## Abstract

This study aimed to examine differences in all-cause mortality and main causes of death across different migrant and local-born populations living in six European countries. We used data from population and mortality registers from Denmark, England & Wales, France, Netherlands, Scotland, and Spain. We calculated age-standardized mortality rates for men and women aged 0–69 years. Country-specific data were pooled to assess weighted mortality rate ratios (MRRs) using Poisson regression. Analyses were stratified by age group, country of destination, and main cause of death. In six countries combined, all-cause mortality was lower for men and women from East Asia (MRRs 0.66; 95 % confidence interval 0.62–0.71 and 0.76; 0.69–0.82, respectively), and Other Latin America (0.44; 0.42–0.46 and 0.56; 0.54–0.59, respectively) than local-born populations. Mortality rates were similar for those from Turkey. All-cause mortality was higher in men and women from North Africa (1.09; 1.08–1.11 and 1.19; 1.17–1.22, respectively) and Eastern Europe (1.30; 1.27–1.33 and 1.05; 1.01–1.08, respectively), and women from Sub-Saharan Africa (1.34; 1.30–1.38). The pattern differed by age group and country of destination. Most migrants had higher mortality due to infectious diseases and homicide while cancer mortality and suicide were lower. CVD mortality differed by migrant population. To conclude, mortality patterns varied across migrant populations in European countries. Future research should focus both on migrant populations with favourable and less favourable mortality pattern, in order to understand this heterogeneity and to drive policy at the European level.

**Electronic supplementary material:**

The online version of this article (doi:10.1007/s10654-015-0083-9) contains supplementary material, which is available to authorized users.

## Introduction

Europe is becoming increasingly ethnically diverse, with currently around 9 % of its total populations comprising migrants [1]. The recent *Lancet* series “Health in Europe” specifically addressed the vulnerable health of migrants and the substantial health inequalities, arguing that political attention is needed at the European level [2].

Country-level analyses from several European countries have demonstrated differences in all-cause mortality between migrant and local-born populations. For example, a comprehensive Dutch study showed that almost all migrant populations have higher all-cause mortality than local-Dutch [3]. However, such country-level studies provide an incomplete picture of migrant health in Europe. It has been shown that migrants originating from specific countries settle across different European countries [1]. Further, each European country has its own unique mix of migrants in terms of country of birth [1].

Cross-national overviews may capture migrant mortality in Europe more comprehensively, and are therefore needed to drive policy at the European level. To fill this gap in the literature, the Migrant and Ethnic Health Observatory (MEHO) project collected cross-national data on specific mortality causes across migrant populations living in Europe [4]. It showed that circulatory disease mortality varied by country of birth in each of the six European countries, with some populations having an excess mortality (e.g., South Asians and Eastern Europeans) [5]. This project also found that diabetes mortality was higher in migrant populations than the local-born populations, especially among migrants from low-income countries, suggesting that socioeconomic change might play a role [6]. Another study showed that cancer mortality was consistently lower in Turkish compared with the local-born populations in four European countries [7].

The present study builds on this work by assessing all-cause mortality and main causes of death for different migrant populations living in six European countries using data from the MEHO project. Such overviews are lacking in Europe so far. This overview helps identify which migrant populations have higher mortality risk and which ones have lower risk in Europe. This could be useful for setting priorities in research and health policy, so that health equity can be achieved in a diverse Europe.

## Methods

### Study design

We used data from the MEHO project. Full details on the data acquisition of this project have been reported elsewhere [4].

We obtained all-cause and cause-specific mortality data by country of birth, sex, and age. Data were drawn from six European countries: Denmark, England & Wales (E&W), France, Netherlands, Scotland, and Spain. For Denmark and the Netherlands, data was derived using linkages between records of the population register and subsequent mortality data. Since an open cohort design was used, participants could enter and exit the study at any point in time during the follow-up period. For E&W, France, Scotland, and Spain, unlinked data was used. We derived numbers of deaths by country of birth, sex, and age from the national mortality registers, and calculated the corresponding person-years at risk (PYR) using population census information. Time periods of data collection varied across countries of destination (see Table 1). Data was anonymised, so no ethical approval was needed.

**Table 1:** Person-years at risk (PYR), total deaths, and age-standardized mortality rate (ASMR) by region of origin in six European countries

Country of destination/region of origin	PYR		Total deaths (n)		ASMR (per 100,000 PY)	
	Men	Women	Men	Women	Men	Women
Denmark, linked data, 1992–2001						
Local-born	23,746,475	23,180,331	104,682	69,666	347.7	221.4
North Africa^a^	28,302	22,682	79	19	545.0	190.5
Sub-Saharan Africa^b^	59,569	55,858	63	47	263.2	119.7
South Asia^c^	94,702	84,447	299	131	562.6	297.8
East Asia^d^	64,856	104,051	132	122	308.3	214.6
Eastern Europe^e^	220,385	226,249	638	456	484.9	257.7
Turkey	182,159	161,540	522	227	607.0	324.7
England & Wales, unlinked data, 1999–2003						
Local-born	102,407,760	102,284,340	338,241	215,470	258.4	157.8
North Africa	158,157	108,510	555	261	298.9	178.5
Sub-Saharan Africa	1,641,200	1,715,870	4524	2872	255.1	160.6
Caribbean^f^	492,580	605,262	3692	2483	308.4	175.4
South Asia	2,271,330	2,259,465	9405	5303	255.3	155.4
Eastern Europe	285,077	421,770	1317	906	325.7	146.8
France, unlinked data, 2005–2007						
Local-born	71,477,377	72,170,529	245,568	114,830	280.5	123.0
North Africa	3,445,897	3,184,913	15,900	6676	212.9	103.9
Sub-Saharan Africa	1,171,073	1,179,280	3481	1894	241.5	161.5
Caribbean	47,279	57,864	84	63	182.4	99.0
Other Latin America^g^	155,728	201,026	235	157	171.2	81.6
South Asia	53,573	49,359	134	49	235.2	132.3
East Asia	373,526	418,176	1183	599	192.2	96.2
Eastern Europe	374,582	484,253	1783	842	331.0	127.8
Turkey	372,175	317,687	755	250	186.2	84.0
The Netherlands, linked data, 1996–2006						
Local-born	65,253,814	63,575,710	215,125	135,367	249.3	155.7
North Africa	1,612,176	1,431,578	2702	1267	207.6	136.5
Caribbean	2,227,202	2,347,797	5540	3412	337.1	185.5
Turkey	1,848,426	1,690,786	3851	1618	295.7	147.7
Scotland, unlinked data, 1999–2003						
Local-born	10,677,105	10,955,370	51,270	32,223	364.3	208.2
North Africa	8210	4690	34	16	459.6	293.8
Sub-Saharan Africa	44,995	44,920	122	82	350.8	223.1
Caribbean	3515	4510	11	11	239.8	160.6
Other Latin America	5,985	7,540	8	9	108.3	87.8
South Asia	80,925	80,355	289	173	240.0	159.0
East Asia	28,695	30,265	72	38	214.5	124.5
Eastern Europe	8,080	11,765	47	24	558.8	183.4
Spain, unlinked data, 2001–2005						
Local-born	86,293,659	85,302,035	297,428	126,250	264.8	109.8
North Africa	1,504,571	781,205	3421	1230	298.5	169.9
Sub-Saharan Africa	358,120	167,478	872	259	412.1	210.1
Caribbean	304,863	469,072	537	432	210.6	100.6
Other Latin America	2,506,164	2,831,886	2941	1869	170.7	89.1
East Asia	137,002	124,429	130	64	120.8	71.5
Eastern Europe	885,962	774,551	2017	697	318.5	143.1

*PYR* Person-years at risk, *ASMR* age-standardized mortality rate

^a^Including migrants from Algeria, Morocco, Tunisia, and other Northern Africa

^b^Ivory Coast, Democratic Republic Congo, Madagascar, Mail, Cameroun, Congo, Senegal, Somalia, South Africa, East Africa, West Africa, Central Southern Africa, and other Africa

^c^Pakistan, Sri Lanka, India, Indian & Southern Oceans, and Bangladesh

^d^South Eastern Asia, Cambodia, China, Japan, Thailand, and Vietnam

^e^Albania, Bosnia-Herzegoniva, Serbia and Montenegro, Yugoslavia, Romania, Bulgaria, Poland, Russia, Ukraine, and other East Europe

^f^The Caribbean countries and Haiti

^g^Brazil, South America, Central-South America, Central America, and other Latin America

### Variables

All-cause mortality data was defined as death from any cause. Depending on the country of destination, we employed either the 9th or 10th revision of International Classification of Diseases (ICD). For main causes of death, we included only the causes that constituted at least 1 % of the total deaths in the majority of the migrant populations (see Online Resource 1 for the prevalence rates of the causes of death by region of birth). We chose the following major groupings (ICD codes in brackets): infectious diseases (ICD-9 279.5, 001–139; ICD-10 B20–B24, A00–B99), cancer (ICD-9 140–239; ICD-10 C00–D48), cardiovascular diseases (CVD) (ICD-9 390–459; ICD-10 I00–I99), diabetes (ICD-9 250; ICD-10 E10–E14), and injuries (ICD-9 E800–999; ICD-10 V01–Y98). Given the expected heterogeneity within these groupings, we examined specific causes of death within the cancer and injuries, and infectious diseases groupings. Specific causes of deaths within CVD have been studied earlier [4, 5, 8]. We examined the following causes of cancer mortality (ICD codes in brackets): oesophagus and oral cavity (ICD-9 140–150; ICD-10 C00–C15), stomach (ICD-9 151; ICD-10 C16), colon/rectum (ICD-9 153–154; ICD-10 C18–C21), liver (ICD-9 155; ICD-10 C22.0–C22.1), breast (ICD-9 174–175; ICD-10 C50), Hodgkin’s disease and leukaemia (ICD-9 201, 204–208; ICD-10 C81, C91–95), and lung and bronchus (ICD-9 161–163, 165; ICD-10 C30–34, C39). For injury-related mortality, the following causes were assessed: unintentional injuries (ICD-9 E800–E915; ICD-10 V01–V99, W00–X59), suicide (ICD-9 E950–959; ICD-10 X60–X84, Y87.0), and homicide (ICD-9 E960–E969; ICD-10 X85–Y09, Y87.1). For infectious diseases, we assessed TB (ICD-9 279.5 and HIV/AIDS (B20–B24).

We focused on migrant populations originating from outside Western Europe and the OECD countries. Given the different migration and colonial history, the size of populations by country of birth differed across European countries. Hence, we categorized the migrant populations into larger geographical regions of birth, to make the findings comparable across countries. This classification was based on the Global Burden of Diseases 2010 study [9]. The following region-of-origin groupings were included: North-Africa, Sub-Saharan Africa, the Caribbean, Other Latin America, South Asia, East Asia, Eastern Europe, and Turkey. Since Turkey cannot be easily confined to a particular region (due to its geographical location and historical links with Europe), it was not included as part of a larger regional grouping but was analysed separately. The local-born population of each European country was also included. Region-of-origin groupings with very few deaths and a relatively low PYR (<3000) were excluded from analysis.

Age was categorized into five-year age groups. We restricted the analysis to those aged 0–69 years because there were very few deaths at age 70+ years for most migrant populations. In the linked data (i.e., longitudinal), the age of participants was recorded at baseline, and participants were followed for the duration of the study, irrespective of their age at baseline. Hence, the participants were not censored according to age. In the unlinked data (i.e., cross-sectional), age was recorded in the mortality registry as the age of death; all deaths among people of 70+ years were excluded.

### Data analysis

We calculated the age-standardized mortality rates (ASMR) based on direct standardisation using the WHO World Standard Population [10].

To assess the differences in all-cause mortality between migrant and local-born populations in Europe, we pooled the data from the six countries. We created region-of-origin-, sex-specific weights which were inversely proportionate to the number of PYR of each region of origin and sex. These weights were assigned to the individual observations based on the region of origin and sex. Weighted mortality rate ratios (MRRs) were computed using Poisson regression, with the local-born populations as the reference group. All models used numbers of deaths (all-cause or cause-specific) as the dependent variable, with five-year age groups, sex, and region of origin as the independent variables, and PYR as the offset variable. MRRs were considered significant at *p* value <0.05.

We first used sex-specific models to assess differences in all-cause mortality in six European countries combined. We then stratified the analyses by age group (0–19, 20–44, and 45–69), country of destination, and main causes of death. However, because of small numbers of deaths for some populations, cause-specific models were not stratified by sex. We used IBM SPSS version 21.0 for analysis.

## Results

Table 1 presents the total deaths and ASMRs by sex, country of destination, and region of origin. Overall, ASMRs for men were between 108.3 and 607.0 and for women between 71.5 and 324.7. For example, Turkish men and women in Denmark had ASMRs of 607.0 and 324.7, respectively, and those in France had ASMRs of 186.2 and 84.0, respectively. For men and women from North Africa the ASMRs varied between 212.9–545.0 and 103.9–293.8, respectively.

Table 2 shows the MRRs in all-cause mortality by region of origin and sex in six countries combined, compared with the local-born populations. MRRs were lower in men and women from East Asia (0.66; 95 % confidence interval [CI] 0.62–0.71 and MRR 0.76; 95 % CI 0.69–0.82, respectively), and Other Latin America (0.44; 0.42–0.46 and 0.56; 0.54–0.59, respectively). Conversely, higher all-cause mortality was observed in women from Sub-Saharan Africa (1.34; 1.30–1.38), and men and women from North Africa (1.09; 1.08–1.11 and 1.19; 1.17–1.22, respectively) and Eastern Europe (1.30; 1.27–1.33 and 1.05; 1.01–1.08 respectively).

**Table 2:** Mortality rate ratios (MRRs) for all-cause mortality in six European countries combined, by region of origin and sex

Region of origin	MRRs* (local-born = 1, ref.)			
	Men	95 % CI	Women	95 % CI
North Africa	**1.09**	**1.08–1.11**	**1.19**	**1.17–1.22**
Sub-Saharan Africa	0.98	0.95–1.00	**1.34**	**1.30–1.38**
Caribbean	**0.85**	**0.83–0.87**	0.97	0.94–1.00
Other Latin America	**0.44**	**0.42–0.46**	**0.56**	**0.54–0.59**
South Asia	**0.91**	**0.89–0.94**	**0.93**	**0.90–0.96**
East Asia	**0.66**	**0.62–0.71**	**0.76**	**0.69–0.82**
Eastern Europe	**1.30**	**1.27–1.33**	**1.05**	**1.01–1.08**
Turkey	**0.96**	**0.93–0.99**	0.96	0.91–1.01

Bold indicates statistical significance at *p* < 0.05

* Adjusted for age and country of destination

### Stratified by age groups

In Table 3 age-specific analyses are presented for different migrant populations, compared with local-born populations of the same age group. Those from North Africa, South Asia, and Turkey had higher all-cause mortality in the age group 0–19 years, in contrast to the other age groups. Specifically, those from South Asia mortality had higher in the age group 0–19 years but lower in the other age groups. For women from Sub-Saharan Africa, all-cause mortality was higher in all age groups, while for men mortality was more or less similar in all age groups. Those from Eastern Europe (especially men) had higher mortality in the age groups 20–44 and less so in 45–69 years, with no differences in the youngest age group.

**Table 3:** Mortality rate ratios (MRRs) in six European countries combined, by region of origin, age group and sex

Region of origin	MRRs* (95 % CI) (local-born = 1, ref.)					
	Men			Women		
	0–19 years	20–44 years	45–69 years	0–19 years	20–44 years	45–69 years
North Africa	**2.20 (2.09–2.31)**	**1.06 (1.03–1.09)**	**1.03 (1.01–1.05)**	**1.11 (1.02–1.20)**	**1.57 (1.50–1.63)**	**1.09 (1.07–1.12)**
Sub-Saharan Africa	1.01 (0.91–1.11)	1.00 (0.97–1.04)	**0.94 (0.92–0.97)**	**1.45 (1.31–1.60)**	**1.59 (1.52–1.67)**	**1.19 (1.15–1.24)**
Caribbean	**0.49 (0.40–0.61)**	**0.98 (0.94–1.03)**	**0.82 (0.80–0.84)**	**0.27 (0.18–0.40)**	**1.26 (1.18–1.34)**	**0.92 (0.89–0.95)**
Other Latin America	**0.54 (0.44–0.65)**	**0.60 (0.56–0.65)**	**0.38 (0.36–0.40)**	**0.23 (0.15–0.36)**	**0.82 (0.76–0.89)**	**0.49 (0.47–0.52)**
South Asia	**1.62 (1.45–1.82)**	**0.84 (0.80–0.89)**	**0.91 (0.89–0.94)**	**1.40 (1.20–1.63)**	**0.92 (0.85–0.99)**	**0.91 (0.87–0.95)**
East Asia	1.00 (0.75–1.35)	**0.60 (0.52–0.69)**	**0.67 (0.61–0.73)**	0.71 (0.49–1.01)	**0.75 (0.64–0.88)**	**0.77 (0.70–0.86)**
Eastern Europe	1.01 (0.88–1.16)	**1.59 (1.51–1.67)**	**1.24 (1.21–1.28)**	0.92 (0.78–1.09)	**1.09 (1.02–1.16)**	1.03 (1.00–1.07)
Turkey	**1.62 (1.46–1.79)**	**0.69 (0.64–0.74)**	1.01 (0.97–1.05)	**1.48 (1.30–1.69)**	**0.77 (0.69–0.86)**	0.98 (0.92–1.05)

Bold indicates statistical significance at *p* < 0.05

* Adjusted for age and country of destination

### Stratified by country of destination

**Fig. 1:**
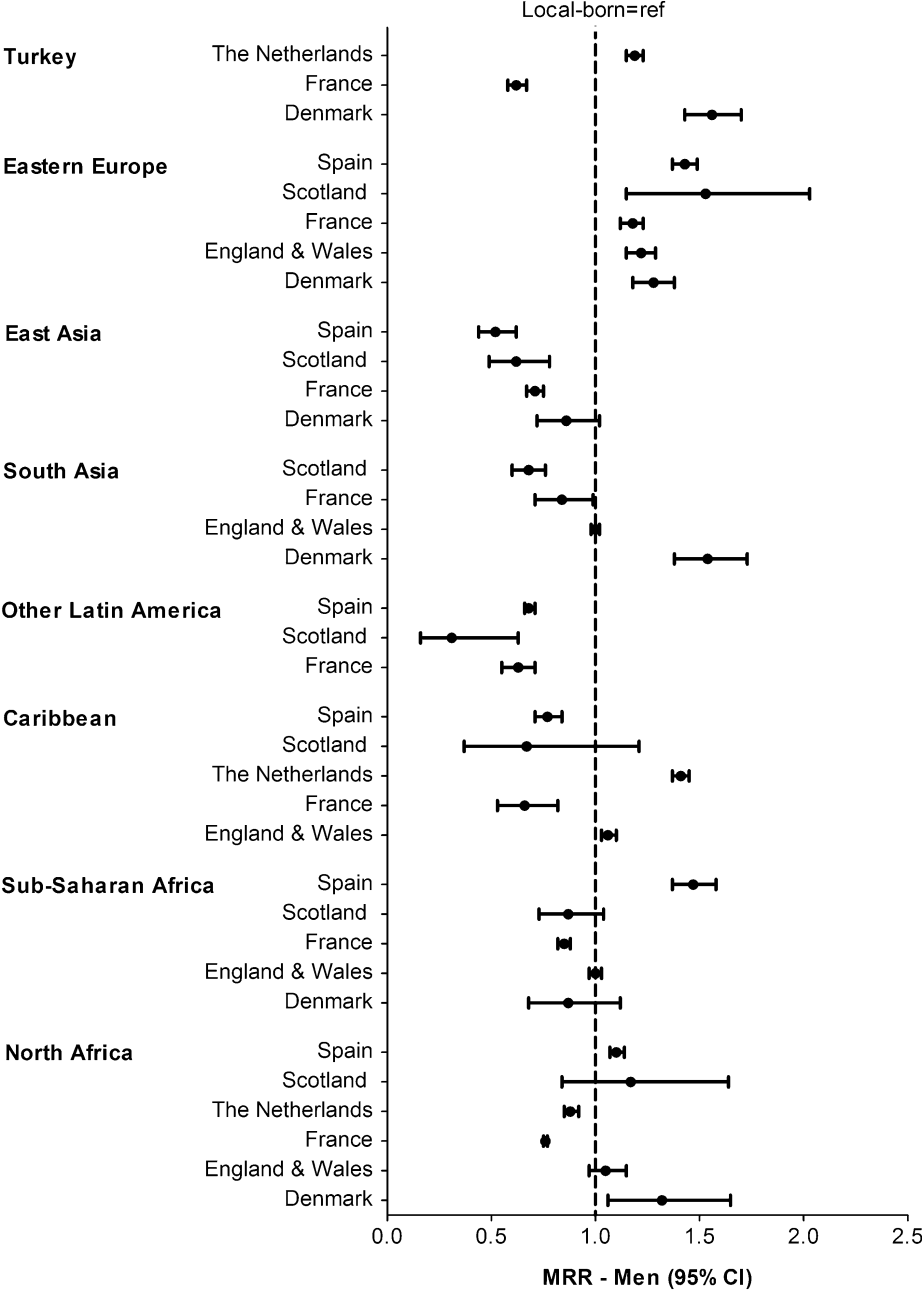
Age-adjusted mortality rate ratios (MRRs) in men by region of origin and country of destination, with local-born as reference population

**Fig. 2:**
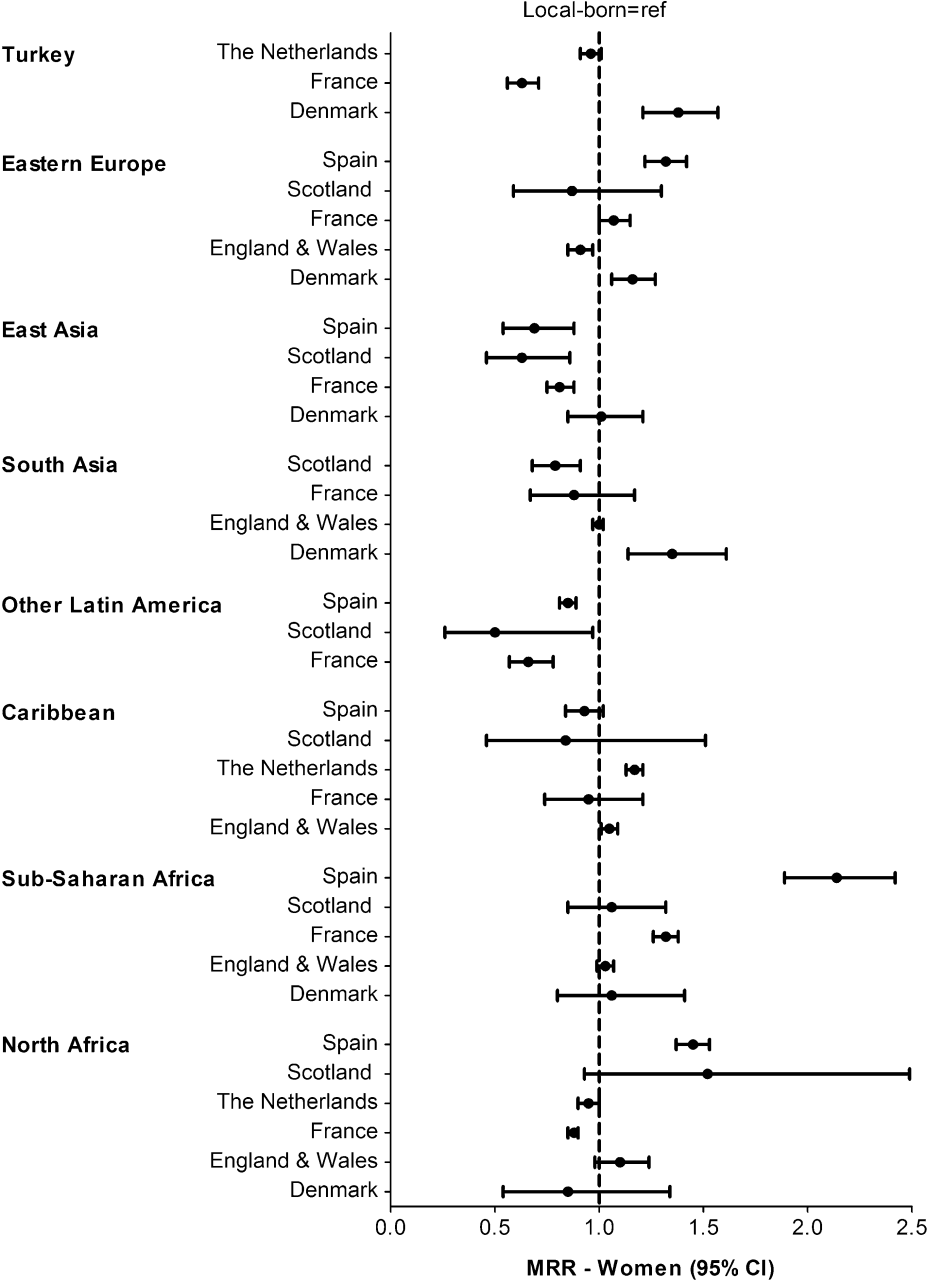
Age-adjusted mortality rate ratios (MRRs) in women by region of origin and country of destination, with local-born as reference population

Compared to the local-born populations, the mortality pattern in different migrant populations varied by country of destination. For men, Eastern Europeans had higher mortality in all European countries. South Asian men had higher mortality in Denmark, similar in E&W, and lower in France and Scotland (Fig. 1). For women, those from North Africa tended to have higher mortality in Spain, Scotland and E&W, similar in Denmark, and lower in the Netherlands and France (Fig. 2). East Asian women had lower mortality in Spain, Scotland and France, and similar in Denmark.

### Stratified by causes of death

Cause-specific analyses are shown in Table 4 (see also Supplementary Material), combined for men and women and for six countries. Compared to local-born populations, mortality due to infectious diseases was higher for most migrant populations, but similar for those from East Asia, Eastern Europe and Turkey. The most important causes were TB and HIV/AIDS, with variations between migrant populations. Cancer mortality was generally lower for the migrant populations, but the pattern varied by cancer site. Mortality due to oesophagus/oral cavity, colon/rectum, and lung/bronchus cancer was lower in the migrant populations. Liver cancer mortality was higher for those from North Africa, Sub-Saharan Africa, and especially East Asia. Mortality due to stomach cancer was higher among those from North Africa, Caribbean, Eastern Europe and Turkey. CVD and diabetes mortality tended be higher for most migrant populations, but consistently lower for those from East Asia and Other Latin America. For injury-related mortality a contrasting picture emerged: for most migrant populations suicide was lower, while homicide was higher.

**Table 4:** Mortality rate ratios (MRRs) combined for men and women and for six European countries, by region of origin and cause of death

Cause of death	Region of origin (local-born = 1, ref.)							
	MRR (95 % CI)							
	North Africa	Sub-Saharan Africa	Caribbean	Other Latin America	South Asia	East Asia	Eastern Europe	Turkey
Infectious diseases	2.55 (2.39–2.71)	4.60 (4.34–4.67)	2.48 (2.20–2.79)	1.42 (1.24–1.63)	2.06 (1.85–2.29)	1.04 (0.77–1.41)	0.84 (0.69–1.02)	0.92 (0.75–1.13)
TB	2.73 (2.09–3.57)	9.41 (7.52–11.76)	1.25 (0.69–2.27)	23.05 (19.10–27.81)	19.05 (15.81–22.97)	0.56 (0.08–3.98)	2.28 (1.37–3.79)	3.22 (1.72–6.01)
HIV/AIDS	0.45 (0.37–0.55)	5.52 (5.12–5.94)	9.28 (8.19–10.52)	1.00 (0.72–1.40)	0.71 (0.53–0.94)	0.28 (0.13–0.42)	0.35 (0.22–0.57)	0.24 (0.14–0.42)
Cancer	0.80 (0.78–0.82)	0.95 (0.92–0.99)	0.77 (0.74–0.80)	0.47 (0.44–0.49)	0.53 (0.51–0.56)	0.80 (0.73–0.88)	1.05 (1.01–1.09)	0.67 (0.63–0.71)
Oesophagus and oral cavity	0.33 (0.29–0.38)	0.61 (0.53–0.70)	0.32 (0.27–0.38)	0.04 (0.02–0.08)	0.42 (0.35–0.51)	0.96 (0.69–1.34)	0.70 (0.60–0.82)	0.34 (0.25–0.45)
Stomach	1.44 (1.31–1.59)	1.01 (0.84–1.21)	2.15 (1.95–2.36)	0.28 (0.20–0.40)	0.95 (0.78–1.15)	0.80 (0.50–1.29)	1.97 (1.71–2.27)	1.88 (1.51–2.33)
Colon/rectum	0.64 (0.58–0.70)	0.75 (0.66–0.85)	0.82 (0.74–0.90)	0.74 (0.64–0.85)	0.44 (0.37–0.52)	0.67 (0.48–0.92)	0.76 (0.66–0.86)	0.60 (0.49–0.74)
Liver	2.17 (1.92–2.45)	2.96 (2.68–3.27)	0.96 (0.78–1.19)	0.61 (0.47–0.80)	1.04 (0.84–1.29)	5.09 (3.94–6.57)	0.76 (0.58–0.99)	0.89 (0.69–1.15)
Breast	0.90 (0.84–0.97)	1.45 (1.33–1.58)	0.67 (0.61–0.74)	0.52 (0.45–0.60)	0.61 (0.53–0.71)	0.27 (0.17–0.44)	0.86 (0.78–0.96)	0.41 (0.32–0.52)
Hodgkin’s disease and leukaemia	1.55 (1.41–1.70)	0.86 (0.72–1.03)	1.72 (1.53–1.94)	0.75 (0.59–0.94)	0.43 (0.32–0.58)	1.16 (0.78–1.72)	1.61 (1.39–1.87)	1.47 (1.20–1.80)
Lung and bronchus	0.71 (0.67–0.74)	0.70 (0.65–0.76)	0.73 (0.69–0.78)	0.36 (0.32–0.40)	0.43 (0.39–0.47)	0.69 (0.57–0.84)	1.08 (1.01–1.16)	0.67 (0.60–0.75)
Cardiovascular diseases	1.25 (1.22–1.28)	1.15 (1.10–1.20)	0.78 (0.75–0.81)	0.39 (0.36–0.42)	1.25 (1.21–1.30)	0.63 (0.55–0.71)	1.27 (1.22–1.32)	1.29 (1.21–1.37)
Diabetes	2.12 (1.96–2.29)	1.25 (1.07–1.46)	1.44 (1.27–1.63)	0.27 (0.19–0.38)	1.89 (1.66–2.15)	0.33 (0.17–0.66)	1.25 (1.06–1.47)	1.96 (1.65–2.33)
Injury-related	1.06 (1.02–1.09)	0.79 (0.75–0.82)	1.05 (1.00–1.10)	0.68 (0.63–0.72)	0.61 (0.57–0.65)	0.74 (0.65–0.84)	1.90 (1.82–1.98)	0.52 (0.48–0.57)
Unintentional injuries	1.22 (1.17–1.27)	0.86 (0.81–0.91)	1.43 (1.35–1.52)	0.94 (0.87–1.01)	0.60 (0.55–0.66)	0.77 (0.66–0.91)	2.25 (2.13–2.37)	0.52 (0.47–0.58)
Suicide	0.60 (0.56–0.64)	0.57 (0.52–0.62)	0.52 (0.46–0.58)	0.36 (0.32–0.42)	0.55 (0.49–0.62)	0.51 (0.39–0.67)	1.42 (1.31–1.54)	0.43 (0.37–0.50)
Homicide	2.83 (2.56–3.14)	1.78 (1.51–2.10)	0.84 (0.62–1.13)	0.39 (0.26–0.59)	1.35 (1.08–1.68)	1.89 (1.44–2.48)	1.52 (1.18–1.96)	1.89 (1.44–2.47)

MRRs were adjusted for sex, age, and country of destination

*TB* tuberculosis, *HIV* human immunodeficiency virus, *AIDS* acquired immune deficiency syndrome

## Discussion

This overview showed a heterogeneous pattern in differences in all-cause mortality between migrant and local-born populations in six European countries. Some migrant populations had lower mortality than local-born populations (e.g., those from East Asia), while others had similar (e.g., Turkey) or higher mortality (e.g., Eastern Europe). The pattern varied importantly by age group, country of destination, and main cause of death. Those from South Asia and Turkey had higher mortality only in the age group 0–19 years, while those from Eastern Europe had higher mortality particularly in the age group 20–69 years. Eastern European men had higher mortality in all European countries, but other migrant populations had a diverse mortality pattern across countries of destination. Generally, most migrant populations had higher mortality due to infectious diseases, and homicide, but lower mortality due to cancer and suicide. CVD mortality differed by migrant population.

This study is the first to assess difference in all-cause mortality and main causes of death between migrant and local-born populations across Europe. It provides a detailed picture of mortality differences among migrant populations living in European countries. However, there are several limitations. First, data collection methods differed across countries, with some using linked data and others unlinked. Arguably, unlinked data are prone for numerator/denominator bias due to differences in the recording of the country of origin between the mortality register (numerator) and the population census (denominator) [6]. This bias may also arise when migrants are included in population censuses but not in mortality registers, or vice versa. This might occur especially in countries where migrants have no incentives to register themselves in population censuses but are recorded when they die. Such bias may depend on incentives to register; for example, being eligible for primary care and preventive services. As such, this bias may differ across countries of destination, and its strength may differ according to age, sex, and migrant population.

Second, the time periods for data collection varied. More specifically, both the length of the time-periods and the time when the data was collected varied. Third, the death registration systems (including death certification, coding practices, ICD coding version, etc.) differed by country of destination, potentially leading to differences in the classification of some causes of death (e.g., diabetes). Finally, because of return migration to their country of birth [11], under-registration of deaths among some migrant populations might have occurred, thereby resulting in an underestimation of all-cause mortality.

An important question is how much of the observed differences in mortality could be attributed to socio-economic position of migrants. While it is unlikely that socio-economic factors are responsible for the lower mortality rates that are observed for migrant populations and for several causes of death, they may have contributed to higher mortality [3, 12]. Unfortunately, we could not assess the role of such factors because individual-level socio-economic variables were not available in a comparable way in the data for the different countries.

Our findings are consistent with previous European country-level studies, which found that all-cause mortality differed across migrant populations. For example, in the Netherlands, all-cause mortality was higher for those from the Caribbean and Turkey, and lower for those from Morocco [3]. In E&W, a similar diverse pattern was observed, with some migrant populations having higher mortality (e.g., Indian women) and others lower (e.g., East Asian men) [13, 14]. Our study is not consistent with research from the United States, which consistently shows that migrant populations have lower all-cause mortality. For example, a recent meta-analysis using 58 longitudinal studies found that Hispanics had nearly 18 % lower all-cause mortality risk than white Americans [15]. Two other large studies showed that black (not African Americans), Hispanic and Chinese migrants had lower all-cause mortality than local-born whites [16, 17].

This overview showed that for the same migrant population the relative mortality level varied by country of destination. Apart from data artefacts, such cross-country difference may reflect differences in the composition of that specific migrant population. For example, Caribbeans settled in E&W are mainly from the English-speaking Caribbean, those in France from Haiti, while those in the Netherlands originate from Antilles and Suriname. Additionally, the reason for migration for migrant populations differs by country of destination. Generally, most migrants from Sub-Saharan Africa residing in the E&W arrived as students (or as highly-skilled migrants), while those living in Spain entered Europe as refugees [1].

There were important differences in the mortality pattern across countries of destination. We observed that most migrant populations (particularly men) had lower all-cause mortality than the local-born population in France. In contrast, in Denmark four (out of six) migrant populations among men had higher mortality, a finding that is particularly interesting as the mortality rate of the local-born population is among the highest when compared to other local-born populations. These disparate findings might be explained by differences in measurement (e.g., study period, data collection method) but also by differences in migrant history, socioeconomic position and the extent of salmon bias. Indeed, a recent study showed that the possible effect of salmon bias is small in Denmark [11].

We found that Eastern Europeans (especially middle-aged men) consistently had higher all-cause mortality than local-born populations in Europe. It could be argued that the “healthy migrant effect” (i.e., individuals with relatively “good” health are more likely to migrate) might be less relevant for Eastern Europeans. Indeed, given the free labour movement across the European Union (EU) and the geographical proximity to Western European countries, migration from Eastern Europe is relatively easier and therefore possibly less selective, compared to other regions. The higher all-cause mortality among Eastern Europeans is mainly driven by the higher CVD and injury-related mortality—the latter is possibly linked with hazardous jobs. A Dutch report, for example, showed that Eastern Europeans mainly work in the construction industry with temporary contracts [18]. If this also applies to other Western European countries, then arguably the higher injury-related mortality could be due to a higher exposure to hazardous work environments. Further, as mortality due to alcohol consumption and smoking is relatively high in Eastern Europe [19, 20], one could argue that these health behaviours might also be prevalent among Eastern European migrants, leading to higher CVD mortality. High alcohol consumption is also related to higher injury-related mortality [21].

Conversely, East Asians consistently had lower all-cause mortality than local-born populations. Their relative health advantage might be related to the strong healthy migrant effect, as it is more difficult for East Asians to migrate to Western Europe because of the greater geographical distance. The relatively favourable health behaviours [22, 23] and SES of East Asians should also be explored as a potential explanation for understanding the advantageous mortality pattern as compared to other migrant populations. As suggested by a 2011 report on Chinese (the largest East Asian group) in the Netherlands [24], East Asians generally tend to fare better socioeconomically than other migrant populations, both in terms of educational attainment and employment.

In conclusion, this overview showed a heterogeneous pattern of differences in all-cause and cause-specific mortality between different migrant and local-born populations in Europe. The pattern varied by sex, age group and country of destination. This heterogeneity clearly implies that migrant health is not necessarily worse in Europe. Indeed, there are some migrant populations that are faring particularly well (e.g., East Asia). Further research should help to understand the favourable mortality pattern among these migrant populations, thereby considering the role of selection factors (e.g., healthy migrant effect, salmon bias) and social determinants. Special attention is also needed for migrant populations with relatively high mortality, such as those from Eastern Europe and younger-aged migrants from North Africa, South Asia, and Turkey. This overview substantiates earlier calls [4, 25] as it clearly underlines the need for setting up valid surveillance systems on mortality across Europe, so that cross-country analysis can be conducted. This overview helps us setting priorities in research and health policy at the European level.

## Electronic supplementary material

Below is the link to the electronic supplementary material.

Supplementary material 1 (DOC 55 kb)

## Abbreviation list

ASMR: Age-standardized mortality rate
MRR: Mortality rate ratio
CI: Confidence interval
PYR: Person-years at risk
MEHO: Migrant and Ethnic Health Observatory
CVD: Cardiovascular diseases
SES: Socioeconomic status
E&W: England & Wales
EU: European Union
