# Supplementary material for: All-cause and cause-specific mortality of different migrant populations in Europe
Source: Eur J Epidemiol. 2015 Sep 11;31:655–65. doi: 10.1007/s10654-015-0083-9 (PMC4977342; doi:10.1007/s10654-015-0083-9)
Supplement: Supplementary file 1 — Supplementary material 1 (DOC 55 kb) [file 10654_2015_83_MOESM1_ESM.doc]

**Article title:** All-cause and cause-specific mortality of different migrant populations in Europe

**Journal name:** European Journal of Epidemiology

**Author names:** Umar Z Ikram; Johan P Mackenbach; Seeromanie Harding; Grégoire Rey; Raj S Bhopal; Enrique Regidor; Michael Rosato; Knud Juel; Karien Stronks; Anton E Kunst

**Corresponding author:** Umar Z Ikram, Academic Medical Center, University of Amsterdam, [u.ikram@amc.uva.nl](mailto:u.ikram@amc.uva.nl)

**Supplementary Table: Percentages* and absolute numbers of cause-specific deaths in six European countries combined**, by region of birth

|  | Region of birth | | | | | | | | |
| --- | --- | --- | --- | --- | --- | --- | --- | --- | --- |
| Cause of death | Local-born | North Africa | Sub-Saharan Africa | Caribbean | Other Latin America | South Asia | East Asia | Eastern Europe | Turkey |
|  | % (n) | % (n) | % (n) | % (n) | % (n) | % (n) | % (n) | % (n) | % (n) |
| **All-cause mortality** | 100 (1 946 120) | 100 (32 160) | 100 (14 216) | 100 (16 265) | 100 (5219) | 100 (15 783) | 100 (2340) | 100 (8727) | 100 (7223) |
| **Infectious diseases** | 1.5 (28 608) | 2.7 (857) | 6.1 (869) | 2.6 (431) | 4.5 (234) | 1.1 (181) | 4.3 (101) | 1.4 (126) | 2.2 (156) |
| TB | 0.1 (1426) | 0.2 (67) | 0.7 (104) | 0.2 (25) | 0.2 (10) | 0.9 (147) | 0.3 (6) | 0.2 (21) | 0.1 (5) |
| HIV/AIDS | 0.6 (11373) | 0.8 (247) | 4.0 (575) | 1.0 (166) | 2.9 (149) | 0.1 (16) | 0.9 (20) | 0.3 (22) | 0.2 (16) |
| **Cancer** | 36.1 (701 612) | 40.0 (12 868) | 24.6 (3502) | 21.1 (3440) | 30.1 (1570) | 12.5 (1974) | 43.1 (1009) | 25.6 (2236) | 23.2 (1679) |
| Oesophagus and oral cavity | 2.7 (52 592) | 2.0 (648) | 1.7 (241) | 1.2 (188) | 0.6 (29) | 1.5 (233) | 3.1 (72) | 1.3 (116) | 0.6 (45) |
| Stomach | 1.5 (28 464) | 1.8 (567) | 1.2 (174) | 1.8 (296) | 1.9 (98) | 0.9 (141) | 1.5 (34) | 1.6 (140) | 1.8 (130) |
| Colon/rectum | 3.8 (73 739) | 2.9 (948) | 2.2 (313) | 2.4 (396) | 2.0 (106) | 1.7 (261) | 3.5 (81) | 2.1 (179) | 1.3 (91) |
| Liver | 0.9 (17 808) | 1.7 (541) | 2.3 (321) | 0.6 (96) | 1.0 (53) | 0.1 (9) | 6.9 (161) | 0.6 (54) | 0.9 (68) |
| Breast | 3.9 (76 853) | 3.3 (1,053) | 4.4 (630) | 3.2 (517) | 3.0 (159) | 2.8 (437) | 2.5 (58) | 2.6 (225) | 1.6 (117) |
| Hodgkin’s disease and leukaemia | 1.2 (23 731) | 1.5 (481) | 1.5 (215) | 1.1 (186) | 2.4 (124) | 1.2 (182) | 1.6 (37) | 1.1 (98) | 1.5 (107) |
| Lung and bronchus | 10.4 (202 008) | 11.5 (3702) | 4.6 (654) | 4.7 (768) | 5.7 (300) | 3.7 (584) | 9.5 (223) | 7.8 (683) | 6.2 (450) |
| **Cardiovascular diseases** | 21.3 (415 311) | 17.3 (5566) | 19.9 (2831) | 24.8 (4038) | 15.6 (812) | 37.7 (5952) | 17.4 (407) | 20.4 (1782) | 20.7 (1492) |
| **Diabetes** | 1.4 (27 834) | 2.5 (792) | 1.8 (257) | 5.0 (807) | 0.7 (35) | 4.4 (701) | 1.2 (29) | 1.0 (87) | 2.9 (206) |
| **Injury-related** | 9.0 (174 496) | 12.5 (4006) | 8.2 (1164) | 9.7 (1584) | 31.7 (1652) | 3.0 (472) | 12.6 (296) | 22.5 (1964) | 11.4 (820) |
| Unintentional injuries | 5.2 (101 993) | 8.7 (2804) | 5.2 (734) | 4.6 (748) | 23.5 (1226) | 1.4 (217) | 8.2 (191) | 15.6 (1365) | 5.9 (428) |
| Suicide | 3.5 (68 058) | 2.7 (882) | 2.6 (368) | 3.3 (540) | 4.9 (254) | 1.5 (244) | 3.5 (83) | 5.2 (451) | 3.1 (226) |
| Homicide | 0.2 (4 445) | 1.0 (320) | 0.4 (62) | 1.8 (296) | 3.3 (172) | 0.1 (11) | 0.9 (22) | 1.7 (148) | 2.3 (166) |

TB=tuberculosis. HIV=human immunodeficiency virus. AIDS=acquired immune deficiency syndrome.

* Percentages are based on the total deaths within a population
